# Supplementary material for: Measuring how motivation affects information quality assessment: A gamification approach
Source: PLoS One. 2022 Oct 27;17(10):e0274811. doi: 10.1371/journal.pone.0274811 (PMC9612527; doi:10.1371/journal.pone.0274811)
Supplement: Attachment — Submitted filename: Plos-One---Response-to-Reviewers.pdf [file pone.0274811.s001.pdf]

Response to Reviewers

Dear Reviewers,

We've performed major revisions on our initial manuscript and we are sending a revised original research article entitled **Measuring how motivation affects information quality assessment: a gamification approach** for consideration by **PLOS ONE**.

We believe that this revised manuscript is now appropriate for publication by PLOS ONE as it is aligned with the journal's topics and we followed all your comments.

We would like to thank reviewers for their constructive comments and editors for willing to reconsider our manuscript following major revision. We have **completely rewritten the paper** and **considered all shortcomings highlighted by reviewers**. Enclosed are our comprehensive responses to all your comments.

Thank you for your reconsideration of our manuscript following major revision.

Sincerely,

The Authors

Reviewer 1

1. **Reviewer's comment**: R1

   Introduction (1)*: The study focuses on motivation; however, motivation is not defined as a concept.*

   Author's response:

   Thank you for your suggestion. We improved the text accordingly. In **section 1**, we defined the concept of motivation. In **section 2.2**, we further described the motivation and indicated how it can be considered in a gamified process. Please see the answer to the reviewer's remark **R13** as well.

2. **Reviewer's comment**: R2

   Assessing the quality of information (2.1)*: The discussion of what is quality of information; previous works on its various dimensions etc should come before the discussion of its assessment.*

   Author's response:

   We believe that the existing presentation of the research problem already follows the proposed sequence, where the term **information quality (IQ)** is first defined (the second paragraph in **section 1**) and then we provide specifics about the problem of measurability of IQ (third paragraph of **section 1**). In the aforementioned **section 2.1**, we just provide a comprehensive review of related work in assessing the quality of information (in addition to the introduction in **section 1**), while IQ was already defined in previous **section 1**.

3. **Reviewer's comment**: R3

   Assessing the quality of information (2.1)*: For example, it states on l. 117, R. Y. Wang and Strong (1996) defined a set of dimensions and a framework where dimensions are grouped into a hierarchical model of IQ aspects and their criteria.*

   First this citation is old; are there any more newer frameworks? Also, it mentions just one model, are there any more models? Or other IQ dimensions?

   Author's response:

   R. Y. Wang and Strong (1996) developed the initial model and identified IQ dimensions that were then used in the majority of related work. Several authors later investigated this initial set of IQ dimensions and evaluated the degree to which individual dimensions comply with the needs or expectations of users. In our approach, we applied the selected set of four IQ dimensions that were prevalent in related work to enable us to perform a better and more objective comparison of related research. Additional explanation was added in the following **section 2.1**.

4. **Reviewer's comment**: R4

   Gamification and motivation (2.2)*: As I noted above, motivation is not discussed thoroughly; there is a brief argument in this section however motivation needs to be properly discussed and defined. Gamification on the other hand is described in much more detail and its application is justified.*

   Author's response:

   A definition of motivation was included in **section 1** and a description of motivation in the context of gamification was included in **section 2.2**. For comprehensive feedback please see the response to reviewer **R13**.

5. **Reviewer's comment**: R5

   Participants (3.2): *The study run from April 2015 to March 2020. Why so long? Typically, experiments took few weeks/months.*

   Author's response:

   It was ongoing research, where we initially targeted the undergraduate students (as presented in **section 3.2**) and later the study was extended to other participants to enhance the external validity. The experiment presented in this paper is only a small part of a broader information quality research at the authors' research facility, where we currently focus only on a small subset of available data, while the large dataset still has to be analyzed. The initial purpose of the research was multifold, where measuring the effect of motivation using a gamification approach is only one of the goals.

6. **Reviewer's comment**: R6

   Participants (3.2): *l. 255, the participants were 57.6% undergraduate students and 42.4% non-students. More demographics are needed; gender distribution, etc. selection criteria are not clear.*

   Author's response:

   Comprehensive gender distribution with min, max and median values is already given in the second paragraph of section 3.2 (i.e. "There were 30.7% female and 69.3% male, with ages ranging from 15 to 69 with a median value of 20 years old."). Additional information about the selection process in also included is the first paragraph of section 3.2 (i.e. "Initially, we directly targeted undergraduate University students whom we had direct access and then all potential participants by utilising mail and social media campaigns.").

7. **Reviewer's comment**: R7

   Results and discussion (4): *Results are detailed. However, motivation is not clear how it was measured. Since motivation is pivotal to this study; motivation needs much more detailed presentation. For example, Table 1: ICC results in our research, shows Q depending on results; however, later on, it presents Q as motivation: e.g., p. 26 highly motivated users (Q1).*

   Author's response:

   The results of our approach are summarised in **Table 1** and **Figure 2**, where we highlight the **two-fold purpose** of the experiment. The primary goal was (1) to measure the inter-rater reliability agreement in a form of **ICC(2,1)** in evaluating various IQ dimensions, which is depicted in Y-axis in **Figure 2**. The secondary goal (2) was to measure the motivation of the participants in the gamified evaluation process, which is depicted in the X-axis in **Figure 2** by clustering players into four groups **Q**. The involvement of the participants in a form of motivation is measured by game points, related to the performance of players further determined by the number of attempts and predefined level of correctness of a given IQ dimension. The mechanics of points calculation are defined in **Equation 6** in **section 3.3**, where each participant can make multiple attempts to find the object but is according to **Equation 6** motivated to find the correct answer in the minimum number of attempts. Each failed attempt reduces the number of points awarded to the player at a given level and consequently in the game as a whole. The aforementioned results ICC(2,1) and Q are not related in terms that ICC(2,1) focuses on agreement with other raters, while Q focuses on the provided value of IQ dimension by the rater in relation to the predefined level of correctness associated with the dimension.

   Additional information about the motivation of the participants is now included in **section 4.1**.

8. **Reviewer's comment**: **R8**

   Discussion (4.2): *There is a detail discussion but it looks confusing to see Q as motivation; in order to arrive at safe conclusions, motivation concept and measurements needs justification and better explanation.*

   Author's response:

   Details about the two-fold purpose of our experiment with ICC vs. Q are given in response to reviewer **R7**. Further, we additionally defined the concept of motivation in **section 1** (see the response to reviewer **R1**) and in **section 2.2** (see the response to reviewer **R13**). Lastly, we also provided additional details about evaluation mechanics in **section 3.1**.

9. **Reviewer's comment**: **R9**

   Theoretical implications (4.3.1): *Gamification could be a potential methodological contribution; I don't see any theories discussed in the background study to understand how this study makes a theoretical contribution.*

   Author's response:

   The vast majority of previous studies on the measurability of IQ employed the simple approach of identifying article-length content (usually Wikipedia) of various quality and then targeting a specific group of users (e.g. students, professionals etc.) by asking them to rate the quality of the content by various IQ dimensions. Users did not have any incentive to perform at their best, resulting in a very low agreement level of all results obtained in related work and making results nonsignificant. In our approach, we aimed to increase the agreement level by focusing on motivating participants that evaluated the content of various quality.

   To achieve this goal we introduced gamification into information quality research, which has not been employed in previous studies. We have developed a gamification framework that improves existing methodological approaches to measuring information quality. We agree with the reviewer that this is a methodological contribution and the wording in **section 4.3** was improved to reflect this.

Reviewer 2

10. **Reviewer's comment**: **R10**

    Missing citations. Many statements seem very arbitrary and require citations. Below are a few examples of where citations are required:

    - ***Introduction (1)**: "Making the best possible decisions requires information of the highest quality. As the amount of information available grows, it becomes increasingly difficult to distinguish quality from questionable information. The problem of poor information quality can weaken our decision processes, so we need more reliable measures and new techniques to assess the quality of information. Unfortunately, such assessment can itself be very demanding."*
    - ***Introduction (1)**: "The literature has widely adopted a multidimensional view of IQ to support more effortless management of its complexity."*
    - ***Introduction (1)**: "Regardless of the ICC interpretation used, the values reported in recent studies are poor or at best moderate. This demonstrates that reaching consensus among various raters is difficult when measuring IQ."*
    - ***Gamification and motivation (2.2)**: "Gamification (Gameful design) is a concept where we use game-like elements in various systems to increase user participation, motivation, improve engagement, or to retain users continue using the system."*

    There are many similar problems like the ones I listed above. Please check statements that require citations.

    Author's response:

    The references were added to the first, the second and the third paragraph of **section 1** and to the second paragraph of **section 2.2**.

11. **Reviewer's comment**: **R11**

    For grouping motivating participants from less motivating participants, what factors have been considered in addition to the player's obtained scores? Please consider mentioning more about it.

    Author's response:

    Please see the response to reviewer's comment **R7**, where we addressed this issue.

12. **Reviewer's comment**: **R12**

    What are the benefits and rationals behind using short article length for your study? Please consider mentioning more about it.

    Author's response:

    The benefits of shorter content length with information quality assessment are thoroughly presented in one of the previous research (Fidler and Lavbič 2017) that we referenced multiple times in this paper. We also discussed the implications of shorter statements and their information quality in our research in **sections 4** and **5**.

13. **Reviewer's comment**: **R13**

    Motivation is essential, as discussed in the article. It could be of interest to present more about intrinsic and extrinsic motivation and how both can be considered in a gamified process or apps. E.g., how can different game elements satisfy players' intrinsic and extrinsic motivations?

    Author's response:

    Thank you for this remark. We included three additional paragraphs in **section 2.2** to further highlight the role of motivation in gamification. In the second paragraph of **section 2.2**, we added the perspective of self-determination on motivation and provided definitions for intrinsic and extrinsic motivation.

    Further, in the second paragraph, we added one additional section that presents papers from the literature that study how intrinsic and extrinsic motivation are considered in a gamified process.

    The third added paragraph is focused on game elements. We added a definition of game elements and referenced related work that investigates how can different game elements satisfy players' motivation.

    Please also see the response to reviewer's comment **R1** regarding the definition of the concept of motivation.

14. **Reviewer's comment**: **R14**

    ***Method (3)**: Did the gamified process used in experiments designed and developed by the authors? Why was this gamified process and design chosen for the study instead of alternatives?*

    Author's response:

    The tool for the gamification process was developed entirely by the authors. A major effort has been made to define individual game levels that as closely as possible correspond to selected four IQ dimensions. Another challenge was also to prepare hints for each individual level with various levels of correctness to appropriately measure the variation in information quality by a given IQ dimension. Based on these requirements we could not find an appropriate existing solution to support our study, so a custom novel solution (a web game) was developed.

15. **Reviewer's comment**: **R15**

    Limitations of the study should be acknowledged?

    Author's response:

    Thank you for this remark. We agree that limitations have not been acknowledged. We created a new **section 4.5** where limitations are addressed.

16. **Reviewer's comment**: **R16**

    Completeness, accuracy, representation, and objectivity dimensions of IQ should be introduced and defined shortly. After reviewing the paper twice, I cannot get an exact definition of these terms. Your definition may be different from what the paper's readers and I think.

    Author's response:

    The definitions of Information Quality dimensions were added to **section 2.1**.

17. **Reviewer's comment**: **R17**

    ***Abstract/ purpose**: In the following statement, I think the ICC is the abbreviation of Interclass Correlation Coefficient, not inter-rater agreement. Please check and revise it. "Existing research on the measurability of information quality (IQ) has delivered poor results and demonstrated low inter-rater agreement (ICC) in evaluating IQ dimensions."*

    Author's response:

    Revised as suggested by adding the term Intra-Class Correlation to ICC abbreviation.

18. **Reviewer's comment**: **R18**

    Gamification and motivation (2.2): *In the following sentence, I suggest referring Deterding et al., who defined this definition of gamification. I recommend also referring to Pelling in this section, who originally coined the concept of gamification:*

    - *"In the literature, gamification is often defined as the use of game design elements in non-game contexts (Bovermann and Bastiaens 2020)."*
    - *Deterding S, Dixon D, Khaled R, Nacke L. From Game Design Elements to Gamefulness: Defining "Gamification". 2011 Presented at: MindTrek '11: 15th International Academic MindTrek Conference: Envisioning Future Media Environments; September 28-30, 2011; Tampere, Finland p. 9-15. [doi: 10.1145/2181037.2181040]*
    - *Pelling, N. (2011, August 9). The (short) prehistory of "gamification".... Funding Startups (& Other Impossibilities).* https://nanodome.wordpress.com/2011/08/09/the-short-prehistory-of-gamification/

    Author's response:

    Thank you for referring to Deterding et al. and Pelling. Both references are now included in the first paragraph of **section 2.2**.

19. **Reviewer's comment**: **R19**

    Gamification and motivation (2.2): *In the following sentence, replacing "In-game gamification features" with "game elements" is better.*

    - *"In-game gamification features include points, levels, choice elements, progress bars, and leaderboards."*

    Author's response:

    The proposed phrase was considered and replaced.

20. **Reviewer's comment**: **R20**

    Gamification and motivation (2.2): *In the following sentence, for each filed like sports, one or more related studies should be cited, not Hamari 2017*

    - *"It is used in application fields like sports, health, sustainability, education, marketing, and business (Hamari 2017)."*

    Author's response:

    An additional comprehensive review was performed to reference suggested studies that resulted in improvements in the second paragraph of **section 2.2**.

21. **Reviewer's comment**: **R21**

    Gamification and motivation (2.2): *in the following statements, authors should use phrases such as "as far as we now" or "to our knowledge". Authors can not make such a claim unless they have strong evidence and references to support it.*

    - *"Previously, gamification has not been used to improve IQ assessment and inter-rater agreement."*

    Author's response:

    We paraphrased the claim to start with "To our knowledge ...".
