# Supplementary material for: Measuring how motivation affects information quality assessment: A gamification approach
Source: PLoS One. 2022 Oct 27;17(10):e0274811. doi: 10.1371/journal.pone.0274811 (PMC9612527; doi:10.1371/journal.pone.0274811)
Supplement: Attachment — Submitted filename: myreview.plosone12464.r1ok.pdf [file pone.0274811.s002.pdf]

PLOS ONE

Manuscript Number: PONE-D-22-12464
Article Type: Research Article
Full Title: Measuring how motivation affects information quality assessment: a gamification approach
Short Title: Measuring how motivation affects information quality assessment: a gamification approach
Keywords: information quality; motivation; information quality assessment; inter-rater agreement; task-at-hand; gameful design

Abstract: Purpose - Existing research on the measurability of information quality (IQ) has delivered poor results and demonstrated low inter-rater agreement (ICC) in evaluating IQ dimensions. Low ICC could result in a questionable interpretation of IQ. The purpose of this paper is to analyse whether assessors' motivation can facilitate ICC.
Methodology - To acquire the participants' views of IQ, we designed a survey as a gamified process. Additionally, we selected Web study to reach a broader audience. We increased the validity of the research by including a diverse set of participants (i.e. individuals with different education, demographic and social backgrounds).
Findings - The study results indicate that motivation improved the ICC of IQ on average by 0.27, demonstrating an increase in measurability from poor (0.29) to moderate (0.56). The results reveal a positive correlation between motivation level and ICC, with a significant overall increase in ICC relative to previous studies. The research also identified trends in ICC for different dimensions of IQ with the best results achieved for completeness and accuracy.
Practical implications - The work has important practical implications for future IQ research and suggests valuable guidelines. The results of this study imply that considering raters' motivation improves the measurability of IQ substantially.
Originality - Previous studies addressed ICC in IQ dimension evaluation. However, assessors' motivation has been neglected. This study investigates the impact of assessors' motivation on the measurability of IQ. Compared to the results in related work, the level of agreement achieved with the most motivated group of participants was superior.

Review

Introduction	authors	revision
The study deals with the important problem of poor information quality and the difficulties in assessing it. Leaving in an era of too much information and disinformation, this study is timely and very well focused i.e. Measuring the IQ of unstructured data ,especially with inter-rater agreement results using Interclass Correlation Coefficient (ICC) statistics (l.64).	Thank you for your suggestion. We improved the text accordingly. In **section 1**, we defined the concept of motivation. In **section 2.2**, we further described the motivation and indicated how it can be considered in a gamified process. Please see the answer to the reviewer's remark **R13** as well.	Addressed
The study focuses on motivation; however, motivation is not defined as a concept;		
2 Related work		
2.1 Assessing the quality of information		
The discussion of what is quality of information; previous works on its various dimensions etc should come before the discussion of its assessment.	We believe that the existing presentation of the research problem already follows the proposed sequence, where the term **information quality (IQ)** is first defined (the second paragraph in **section 1**) and then we provide specifics about the problem of measurability of IQ (third paragraph of **section 1**). In the aforementioned **section 2.1**, we just provide a comprehensive review of related work in assessing the quality of information (in addition to the introduction in **section 1**), while IQ was already defined in previous **section 1**.	Addressed
For example, it states on l. 117, R. Y.Wang and Strong (1996) defined a set of dimensions and a framework where dimensions are grouped into a hierarchical model of IQ aspects and their criteria.	R. Y. Wang and Strong (1996) developed the initial model and identified IQ dimensions that were then used in the majority of related work. Several authors later investigated this initial set of IQ dimensions and evaluated the degree to which individual dimensions comply with the needs or expectations of users. In our approach, we applied the selected set of four IQ dimensions that	Looks ok now

	were prevalent in related work to enable us to perform a better and more objective comparison of related research. Additional explanation was added in the following **section 2.1**.	
First this citation is old; are there any more newer frameworks? Also, it mentions just one model, are there any more models? Or other IQ dimensions?		
2.2 Gamification and motivation		
As I noted above, motivation is not discussed thoroughly; there is a brief argument in this section however motivation needs to be properly discussed and defined. Gamification on the other hand is described in much more detail and its application is justified.	A definition of motivation was included in **section 1** and a description of motivation in the context of gamification was included in **section 2.2**. For comprehensive feedback please see the response to reviewer **R13**.	Addressed
2.3 Problem statement and proposed solution		
This section is well developed		
3 Method		
3.1 Evaluation mechanics		
3.2 Participants		
The study run from April 2015 to March 2020. Why so long? Typically, experiments took few weeks/months.	It was ongoing research, where we initially targeted the undergraduate students (as presented in **section 3.2**) and later the study was extended to other participants to enhance the external validity. The experiment presented in this paper is only a small part of a broader information quality research at the authors' research facility, where we currently focus only on a small subset of available data, while the large dataset still has to be analyzed. The initial purpose of the research was multifold, where measuring the effect of motivation using a gamification approach is	Addressed

	only one of the goals.	
l. 255, the participants were 57.6% undergraduate students and 42.4% non-students. More demographics are needed; gender distribution, etc. selection criteria are not clear.	Comprehensive gender distribution with min, max and median values is already given in the second paragraph of section 3.2 (i.e. “There were 30.7% female and 69.3% male, with ages ranging from 15 to 69 with a median value of 20 years old.”). Additional information about the selection process in also included is the first paragraph of section 3.2 (i.e. “Initially, we directly targeted undergraduate University students whom we had direct access and then all potential participants by utilising mail and social media campaigns.”).<br>7.	Addressed
3.3 Measuring IQ dimensions: it is detailed and well presented		
4 Results and discussion		
Results are detailed. However, motivation is not clear how it was measured. Since motivation is pivotal to this study; motivation needs much more detailed presentation. For example, Table 1: ICC results in our research, shows Q depending on results; however, later on, it presents Q as motivation: e.g., p. 26 highly motivated users (Q1).	The results of our approach are summarised in **Table 1** and **Figure 2**, where we highlight the **two-fold purpose** of the experiment. The primary goal was (1) to measure the inter-rater reliability agreement in a form of **ICC(2,1)** in evaluating various IQ dimensions, which is depicted in Y-axis in **Figure 2**. The secondary goal (2) was to measure the motivation of the participants in the gamified evaluation process, which is depicted in the X-axis in **Figure 2** by clustering players into four groups **Q**. The involvement of the participants in a form of motivation is measured by game points, related to the performance of players further determined by the number of attempts and predefined level of correctness of a given IQ dimension. The mechanics of points calculation are defined in **Equation 6** in **section 3.3**, where each participant can make	Looks fine now<br>Make sure Figure 2 is visible online because it contains too much information; perhaps you split it in 2/3 parts

	multiple attempts to find the object but is according to **Equation 6** motivated to find the correct answer in the minimum number of attempts. Each failed attempt reduces the number of points awarded to the player at a given level and consequently in the game as a whole. The aforementioned results ICC(2,1) and Q are not related in terms that ICC(2,1) focuses on agreement with other raters, while Q focuses on the provided value of IQ dimension by the rater in relation to the predefined level of correctness associated with the dimension.<br>Additional information about the motivation of the participants is now included in **section 4.1**.	
4.2 Discussion		
There is a detail discussion but it looks confusing to see Q as motivation; in order to arrive at safe conclusions, motivation concept and measurements needs justification and better explanation.	Details about the two-fold purpose of our experiment with ICC vs. Q are given in response to reviewer **R7**. Further, we additionally defined the concept of motivation in **section 1** (see the response to reviewer **R1**) and in **section 2.2** (see the response to reviewer **R13**). Lastly, we also provided additional details about evaluation mechanics in **section 3.1**.	addressed
4.3.1 Theoretical implications		
Gamification could be a potential methodological contribution; I don't see any theories discussed in the background study to understand how this study makes a theoretical contribution.	The vast majority of previous studies on the measurability of IQ employed the simple approach of identifying article-length content (usually Wikipedia) of various quality and then targeting a specific group of users (e.g. students, professionals etc.) by asking them to rate the quality of the content by various IQ dimensions. Users did not have any incentive to perform at their best,	This work is innovative so it is up to the authors to explain how this study makes a theoretical contribution

	resulting in a very low agreement level of all results obtained in related work and making results nonsignificant. In our approach, we aimed to increase the agreement level by focusing on motivating participants that evaluated the content of various quality.<br>To achieve this goal we introduced gamification into information quality research, which has not been employed in previous studies. We have developed a gamification framework that improves existing methodological approaches to measuring information quality. We agree with the reviewer that this is a methodological contribution and the wording in **section 4.3** was improved to reflect this.	
Recommendation		
Major revision based on the comments above		accept
